# Beyond Correlation: Constraint Architecture Explains Proteome–Metabolome Decoupling

**DOI:** 10.3390/ijms27093971

**Published:** 2026-04-29

**Authors:** Kyung-Hee Kim, Byong Chul Yoo

**Affiliations:** 1 Department of Applied Chemistry, School of Science and Technology, Kookmin University, Seoul 02707, Republic of Korea; kyungheekim@kookmin.ac.kr; 2 Antibody Research Institute, Kookmin University, Seoul 02707, Republic of Korea; 3 Diagnostic Research Team, InnoBation Bio R&D Center, Seoul 03929, Republic of Korea

**Keywords:** proteomics, metabolomics, metabolic flux, constraint-based modeling, thermodynamics, redox metabolism, fluxomics, systems biology

## Abstract

Multi-omics technologies enable parallel quantification of proteomic and metabolomic layers, yet enzyme abundance often shows weak or nonlinear correspondence under diverse biological conditions. This apparent discordance has been attributed to both technical limitations—such as dynamic range compression in LC-MS/MS, metabolite derivatization artifacts, and missing values in proteomic measurements—as well as intrinsic biological properties of metabolic network architecture. While technical factors contribute to cross-omic mismatch, accumulating evidence suggests that constraint-driven network behavior plays a major role in shaping this decoupling. Enzyme abundance constrains catalytic capacity; however, realized flux is selected within this capacity under distributed flux control, as formalized by flux control coefficients in metabolic control analysis, and is further modulated by enzyme kinetics (e.g., km and Vmax), post-translational modifications, substrate availability, and thermodynamic constraints. Metabolite pools, in turn, reflect the physicochemical state of the system, while specific metabolites can also act as regulatory effectors that modulate enzymatic activity and cellular signaling. Because metabolic networks are underdetermined, multiple flux configurations can satisfy identical protein abundance and metabolite concentration data. Static cross-layer correlation is therefore insufficient for mechanistic inference. We synthesize biological mechanisms—including post-translational regulation, allostery, thermodynamic buffering, spatial compartmentalization, feedback amplification, and redox gating—that weaken linear abundance–metabolite expectations. We further outline a constraint-based interpretation framework in which proteomics imposes capacity bounds, metabolomics informs reaction directionality and metabolite pool constraints, and flux-informed approaches reduce solution degeneracy by providing additional information on pathway activity. Moving beyond correlation requires integrating perturbation, temporal resolution, and constraint-aware modeling. Proteome–metabolome discordance should therefore be interpreted not as inconsistency, but as indicative of constraint-driven state selection within high-dimensional biochemical systems.

## 1. Introduction: The Promise and Limits of Multi-Omics Integration

The rapid expansion of high-resolution mass spectrometry has enabled large-scale profiling of proteins and metabolites; however, proteomic and metabolomic platforms differ substantially in sample preparation, ionization, fragmentation strategies, and quantification approaches. While current technologies allow the detection of thousands of proteins and hundreds to thousands of metabolites under optimized conditions, routine experimental coverage is often more limited and influenced by platform-specific biases such as dynamic range constraints and ion suppression effects. While mass spectrometry dominates high-resolution metabolomics, nuclear magnetic resonance (NMR)-based approaches also provide complementary quantitative and structural insights [1]. This technological progress has fueled the expectation that multi-omics integration can provide insight into molecular hierarchies linking gene programs and protein networks [2,3,4]; however, such observations are fundamentally associative and do not, by themselves, establish causal relationships without perturbation-based or interventional approaches. In particular, the integration of proteomics and metabolomics has been widely viewed as a direct path toward mechanistic insight in physiology, disease biology, and therapeutic response.

The underlying assumption often remains implicit: metabolic enzymes are proteins; therefore, changes in protein abundance should shape metabolic outputs in a predictable manner, as formalized in metabolic control analysis [5,6] and related systems-level frameworks [7]. From this perspective, proteomics informs catalytic capacity, whereas metabolomics reflects the biochemical state that results from that capacity. Integration thus appears conceptually straightforward—correlate enzyme abundance with metabolite levels, infer pathway regulation, and construct mechanistic models.

Yet, empirical experience across diverse biological systems has repeatedly revealed that concordance between proteomic and metabolomic layers is frequently weak, nonlinear, or context-dependent [8,9,10,11]. Correlation coefficients are often modest (typically in the range of r ≈ 0.2–0.5, depending on the level of analysis and biological context) across diverse biological conditions [8,9,10,11]; pathway enrichment signals may disagree between layers; and in many perturbation experiments, robust metabolite changes occur without corresponding changes in enzyme abundance, or vice versa [12,13,14]. This recurring observation raises a central question: Is proteome–metabolome discordance merely technical noise, or does it reflect fundamental properties of biochemical systems?

This review argues for the latter. While measurement limitations and incomplete coverage contribute to cross-omic mismatch, biological factors are important contributors to proteome–metabolome decoupling [5,6]. The mapping from protein abundance to metabolite pools is mediated by enzyme activity, thermodynamic constraints, and network topology [5,13]. Each of these layers introduces degrees of freedom that weaken simple abundance-based prediction.

We propose a conceptual distinction between catalytic capacity and metabolic state. Catalytic capacity is approximated by proteomic abundance but is further modulated by regulatory and thermodynamic constraints; metabolic state is reflected in metabolite pools, energy charge, redox ratios, and flux distributions [12,15]. These domains are linked but governed by distinct constraint layers and therefore cannot be reduced to one another. Recognizing this distinction reframes integration: rather than seeking strong correlation between layers, we should identify the biological conditions under which correlation is expected—or structurally constrained to fail.

In the sections that follow, we dissect the principal biological mechanisms that generate proteome–metabolome decoupling and propose an interpretation-first framework grounded in flux, thermodynamics, and causal perturbation. However, a systematic framework that explains this decoupling from a constraint-based perspective remains lacking, which this review aims to address.

## 2. Conceptual Framework: Breakdown of the Linear Cascade

A commonly implied chain connects molecular layers:

Protein abundance → Enzyme activity → Pathway flux → Metabolite pools → Phenotype [5,13].

**Figure 1:**
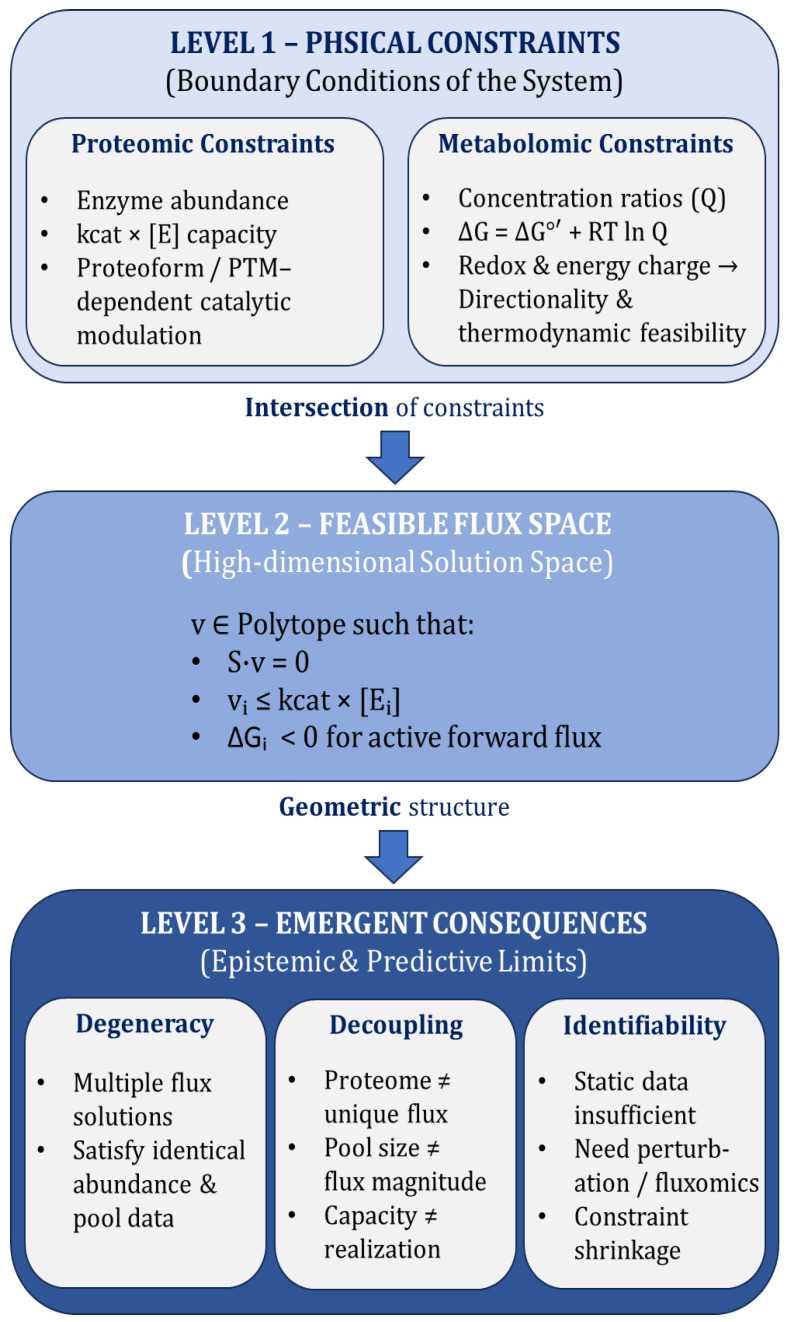
Conceptual illustration of constraint architecture underlying proteome–metabolome decoupling. Proteomic capacity, thermodynamic constraints, and stoichiometric constraints jointly define a feasible space of metabolic flux states. Within this space, multiple flux configurations can satisfy identical proteomic and metabolomic profiles, leading to potential decoupling between molecular layers.

At first glance, this cascade appears logical. However, each transition hides regulatory complexity. Enzyme activity is not uniquely determined by abundance; flux is not uniquely determined by activity; metabolite pools are not uniquely determined by flux; and phenotype often reflects integrated network behavior rather than single pathway throughput [5,6,13,16]. Understanding decoupling requires unpacking each transition (Figure 1). As shown in Figure 1, proteomic capacity, thermodynamic constraints, and stoichiometric relationships define a feasible solution space of metabolic flux states, within which multiple flux configurations can satisfy identical proteomic and metabolomic profiles.

## 3. Mechanistic Layers of Proteome–Metabolome Decoupling

The conceptual framework outlined above provides a basis for understanding how proteome–metabolome decoupling emerges from multiple regulatory layers. In the following section, we examine the specific mechanistic factors that contribute to this decoupling in biological systems. The principal mechanistic layers contributing to proteome–metabolome decoupling are summarized in Table 1.

### 3.1. Post-Translational Regulation

Proteomic datasets typically report aggregate protein abundance without resolving functional proteoforms. However, enzymatic function frequently depends on specific PTMs. Phosphorylation can activate or inhibit catalytic sites; acetylation can alter substrate affinity; redox-sensitive cysteine oxidation can transiently disable enzymes in oxidative environments; ubiquitination can affect stability or localization [25,26].

In metabolic pathways such as glycolysis and the TCA cycle, rapid activity modulation often occurs without measurable changes in total protein abundance. Thus, two samples with identical enzyme abundance can exhibit markedly different catalytic behavior depending on modification state [27,28].

### 3.2. Enzyme Complex Assembly

Some metabolic functions depend on multi-enzyme complexes or metabolons. The abundance of individual subunits does not necessarily reflect the fraction incorporated into functional assemblies. Changes in complex assembly, not total protein amount, may determine pathway throughput [18,29].

### 3.3. Allosteric Feedback

Metabolism is dominated by allosteric regulation. End-products often inhibit upstream enzymes, and energy/redox ratios modulate catalytic rates. These effects operate at timescales far shorter than protein turnover and can reconfigure metabolic output independently of abundance shifts [5,30,31].

## 4. Flux Regulation and Network-Level Constraints

### 4.1. Flux Is a Network Property Rather than a Single-Enzyme Output

A central misconception in multi-omics integration is the implicit assumption that metabolic flux is controlled by one or a few “rate-limiting” enzymes whose abundance directly determines pathway throughput. Although the concept of a rate-limiting step has heuristic value, systems-level metabolic analysis has repeatedly demonstrated that flux control is generally distributed across multiple nodes and varies with system state [5,6,7].

Metabolic control analysis (MCA) formalized this principle by introducing the concept of the flux control coefficient, which quantifies the relative change in pathway flux in response to a relative change in enzyme activity. Importantly, the sum of flux control coefficients across all enzymes in a pathway equals unity under steady-state conditions, implying that control is shared rather than monopolized [6,7].

This framework has several implications for proteome–metabolome integration:

1. Increasing the abundance of a single enzyme does not guarantee a proportional flux increase if that enzyme carries low control under the prevailing conditions.
2. Flux control coefficients are not static; they depend on substrate levels, product inhibition, allosteric regulation, and competing pathways.
3. Control can redistribute dynamically when the system is perturbed.

Therefore, even an accurate measurement of enzyme abundance may provide limited predictive power unless one knows how control is distributed at that specific metabolic state. This alone weakens the expectation of strong static correlation between proteomic and metabolomic layers [5,12,32].

### 4.2. Elasticity, Substrate Sensitivity, and State Dependence

In addition to control coefficients, MCA introduced the concept of elasticity coefficients, which describe how sensitive an enzyme’s reaction rate is to changes in substrate or effector concentration. Elasticity reflects kinetic responsiveness rather than abundance.

An enzyme may be present at high abundance but operate in a regime where its rate is relatively insensitive to substrate concentration (low elasticity), especially if it is near saturation. Conversely, a moderately abundant enzyme operating far from saturation may exert a strong influence over flux because its activity is highly responsive to small substrate changes [6,12,33].

This introduces a key insight: Enzyme abundance sets a ceiling on catalytic capacity, but elasticity and substrate availability determine how much of that capacity is realized.

Consequently, two biological states with identical proteomic profiles can exhibit divergent flux distributions if metabolite concentrations differ. This decoupling arises from state-dependent kinetic properties rather than measurement error.

### 4.3. Reserve Capacity and the Illusion of Rate Limitation

Cells rarely operate metabolic enzymes at maximal velocity under baseline conditions. Many pathways exhibit reserve capacity, meaning that enzyme levels exceed those required to sustain typical flux demands [5,34,35].

This reserve capacity creates buffering. If enzyme abundance increases modestly, flux may not change because the pathway was not limited by that step to begin with. Conversely, flux may increase dramatically when substrate supply changes, even if enzyme abundance remains constant.

The notion of a “rate-limiting enzyme” therefore depends on context. Under one metabolic state, enzyme A may carry substantial control; under another, enzyme B may dominate. Proteomic changes that appear functionally significant in isolation may be absorbed by network buffering mechanisms. This buffering contributes directly to weak cross-omic correlations, as proteome shifts may not alter flux, and flux shifts may occur without corresponding changes in proteome abundance. Reserve capacity buffers flux under baseline conditions, but this buffering can be overcome when substrate availability or network constraints shift.

### 4.4. Network Topology and Alternative Routing

Metabolic networks are highly interconnected. Many intermediates participate in branch points where flux can be rerouted [16,34,35,36,37]. For example, glycolytic intermediates may be diverted toward biosynthesis, redox balancing, or storage pathways depending on cellular demand.

In such networks, increased abundance of one pathway enzyme may not increase net flux through that pathway if alternative routes compensate. Likewise, inhibition of one node may be bypassed by parallel enzymes or isoforms.

This distributed topology further undermines simplistic mapping between enzyme abundance and metabolite pool size. Flux redistribution can stabilize metabolite pools even when underlying enzymatic composition changes.

### 4.5. Nonlinear Amplification and Threshold Effects

Metabolic systems often exhibit nonlinear behaviors such as ultrasensitivity and bistability. Ultrasensitivity can arise when reaction responses display cooperative behavior (e.g., Hill coefficient *n* > 1) or under zero-order conditions in which enzymes operate near saturation [38]. Bistability typically requires the presence of positive feedback loops combined with sufficient nonlinearity in the underlying reaction network [39].

Such behaviors have been observed in metabolic and signaling contexts, including glycolytic oscillations and bistable switches in regulatory networks [38,39,40], where small perturbations in enzyme activity or metabolite levels can trigger disproportionate shifts in system state. In such regimes, static abundance–metabolite correlations can appear weak or discontinuous. The absence of linear correlation does not imply the absence of regulatory influence; it may reflect nonlinear response curves inherent to biochemical networks.

This observation is particularly relevant in disease states and stress responses, where metabolic systems may approach tipping points [13,35].

## 5. Pool–Flux Dissociation and Thermodynamic Constraints

### 5.1. Steady-State Pools Reflect Balance, Not Throughput

At steady state, metabolite concentration is determined by the balance between production and consumption rates. A high turnover metabolite may maintain a low steady-state concentration if its production and removal are tightly matched. Conversely, a bottleneck downstream can cause accumulation even if upstream flux remains unchanged [5,13,20].

Thus, metabolite concentration is not a direct proxy for pathway activity. Instead, it reflects flux imbalance, thermodynamic positioning, compartmental distribution, and transport limitations. This distinction is central to understanding why proteomic abundance often fails to predict metabolomic shifts.

### 5.2. Thermodynamic Feasibility and Near-Equilibrium Reactions

Many metabolic reactions operate near thermodynamic equilibrium. In these cases, enzyme activity has limited influence over metabolite ratios, which are instead governed by Gibbs free energy relationships [13,20,41].

If a reaction is near equilibrium, increasing enzyme abundance can increase catalytic turnover, but net flux in near-equilibrium reactions is primarily determined by the displacement from thermodynamic equilibrium rather than enzyme abundance itself, leaving net flux largely unchanged unless substrate or product concentrations shift. Therefore, proteomic changes may not alter metabolite ratios under equilibrium constraints. This thermodynamic buffering introduces another layer of decoupling between enzyme abundance and metabolite pools.

### 5.3. Energy Charge and Redox Ratios as System-Level Constraints

Cells tightly regulate ATP/ADP/AMP ratios and redox couples such as NAD^+^/NADH and NADP^+^/NADPH. These global state variables constrain metabolic reaction directionality and influence numerous enzymatic steps simultaneously [20,42,43].

When perturbations occur, compensatory flux redistribution often acts to restore these ratios. As a result, metabolite pools may remain stable even when enzyme abundance changes significantly. This systemic homeostasis further weakens simple abundance-based prediction. These compensatory responses may involve mechanisms such as adenylate kinase buffering, AMPK activation, or pentose phosphate pathway induction.

## 6. The Metabolome as a Regulatory Layer

Proteome–metabolome integration is frequently framed as a downstream problem: enzymes produce metabolites, and metabolite levels therefore reflect proteomic composition. This view assumes directional hierarchy. However, biochemical systems do not operate in a strictly feed-forward manner. Metabolites are not merely reaction products; they actively participate in defining catalytic, transcriptional, and thermodynamic architecture [44,45].

The metabolome should therefore be understood not as an endpoint, but as a regulatory layer that both reflects and reshapes enzymatic organization. This regulatory inversion—where metabolites influence the very protein systems that generate them—represents a central source of proteome–metabolome decoupling [46,47]. Three interrelated mechanisms illustrate this inversion.

### 6.1. Epigenetic and Transcriptional Inversion

Several core metabolites directly regulate chromatin-modifying enzymes and transcriptional machinery. Acetyl-CoA supplies acetyl groups for histone acetylation; S-adenosylmethionine provides methyl groups for DNA and histone methylation; α-ketoglutarate serves as a cofactor for dioxygenases involved in demethylation; succinate and fumarate can inhibit these enzymes by competing for binding sites [46,47,48,49].

In such contexts, modest changes in metabolite ratios can reshape chromatin accessibility and transcriptional programs without preceding changes in enzyme abundance. Proteomic remodeling may therefore occur downstream of metabolite-driven epigenetic shifts rather than upstream.

This temporal inversion unfolds in stages:

1. Flux redistribution alters metabolite pools.
2. Metabolites modify chromatin or transcription factors.
3. Gene expression programs adjust.
4. Proteomic composition shifts subsequently.

When sampling captures early phases of this sequence, great metabolomic changes may coexist with minimal proteomic remodeling. Decoupling thus reflects causal ordering rather than analytical inconsistency.

### 6.2. Redox and Chemical-State Regulation

Redox couples such as NAD^+^/NADH and NADP^+^/NADPH operate as global state variables influencing dozens of enzymatic reactions simultaneously. These ratios modulate dehydrogenase activity, regulate sirtuin-dependent deacetylation, and gate signaling cascades sensitive to oxidative status [21,42,50,51].

Because redox variables shift rapidly in response to substrate availability or electron transport constraints, metabolic state can change before proteomic abundance adapts. Enzymes may remain present yet operate under fundamentally altered thermodynamic and redox regimes.

In parallel, non-enzymatic chemical reactions further reshape metabolite composition. Reactive oxygen species oxidize small molecules; lipid peroxidation generates electrophilic species; and spontaneous glycation modifies both metabolites and proteins. These processes include spontaneous oxidation, non-enzymatic glycation, and thermodynamically driven side reactions, which can alter metabolite composition independently of enzyme abundance [52,53,54]. They depend primarily on physicochemical conditions rather than enzyme abundance.

Under such conditions, metabolomic signatures reflect constraint repositioning—particularly redox and energetic state—rather than catalytic scaling. Abundance and state, therefore, diverge.

### 6.3. Direct Metabolite–Protein Regulatory Networks

Beyond epigenetic and redox effects, metabolites frequently bind proteins outside classical catalytic sites, altering stability, localization, and interaction networks [55,56]. These interactions extend regulatory influence beyond substrate–product relationships, embedding metabolites within signaling and structural circuits.

In some biological contexts, accumulation of specific intermediates alters transcription factor stability or enzymatic specificity, thereby reshaping differentiation or growth programs. Such effects often arise from subtle flux redistribution rather than large abundance shifts.

When metabolites regulate enzyme activity, and enzymes in turn regulate metabolite levels, feedback loops emerge. These feedback architectures can generate threshold behavior, bistability, or oscillation. Small perturbations may trigger disproportionate transitions in metabolic state without proportional changes in total protein abundance [39,40]. In feedback-dominated systems, static cross-omic correlation becomes inherently unreliable because catalytic capacity and realized state are co-determined by recursive dynamics.

### 6.4. From Output to State Variable

Taken together, these mechanisms challenge the assumption that metabolites are passive outputs of enzymatic cascades. Instead, metabolite pools function as state variables that define the physicochemical environment within which enzymes operate [13,44].

Enzyme abundance specifies catalytic potential. Metabolite composition defines thermodynamic feasibility, redox balance, and regulatory context. Because these domains interact bidirectionally, neither layer can be reduced to the other [34].

**Figure 2:**
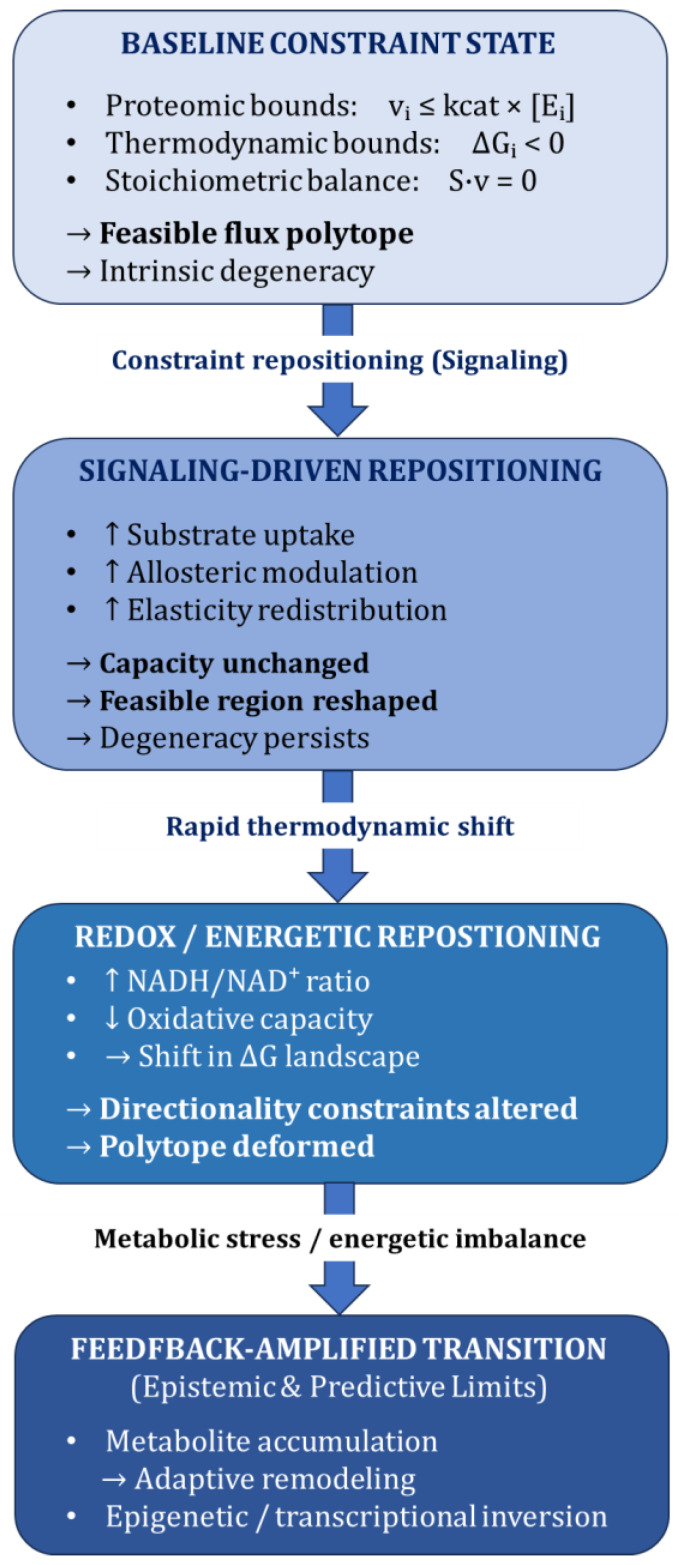
Constraint repositioning and flux state transitions. Dynamic illustration of how signaling, thermodynamic shifts, and metabolic stress reposition the feasible flux space without necessarily altering catalytic capacity. Under baseline conditions, intrinsic degeneracy characterizes the feasible flux polytope. Signaling-driven modulation reshapes the feasible region while preserving overall capacity, allowing degeneracy to persist. Rapid thermodynamic shifts (e.g., altered redox ratios) deform the constraint geometry by modifying ΔG landscapes and reaction directionality. Metabolic stress and energetic imbalance amplify this repositioning, potentially triggering adaptive remodeling. Flux redistribution often precedes large-scale proteomic remodeling, particularly in rapidly changing metabolic states.

This regulatory architecture sets the stage for the next section: when constraint structures shift abruptly—through signaling, redox repositioning, or feedback amplification—the divergence between catalytic capacity and metabolic state becomes particularly visible. The interacting mechanisms that generate such decoupling are summarized in Figure 2.

## 7. Spatiotemporal Dynamics and Constraint-Shifted States

### 7.1. Organelle-Specific Metabolism

Mitochondria, cytosol, nucleus, and peroxisomes maintain distinct metabolite pools and redox states. Transport mechanisms regulate exchange. Bulk measurements obscure these localized differences, masking causality [23,57,58].

### 7.2. Microenvironmental Heterogeneity

Within tissues, cell-type heterogeneity further complicates mapping. Proteomic averages across mixed populations may not align with metabolite pools driven by specific subpopulations [59,60].

In addition to spatial organization, temporal desynchronization further contributes to proteome–metabolome decoupling. Metabolic adjustments often occur within seconds through allostery and substrate shifts, whereas proteomic adjustments require transcriptional and translational changes over hours or days. Single-time-point measurements may therefore capture asynchronous states [9,24,61]. Time-resolved multi-omics approaches are essential to disentangle early metabolic responses from later proteomic remodeling.

Proteome–metabolome decoupling becomes most visible when biological constraints shift abruptly. Rather than treating such contexts as disease-specific phenomena, they can be understood as regimes in which thermodynamic, redox, energetic, or signaling constraints are reconfigured faster than proteomic composition adapts [13,35].

These regimes expose structural properties of metabolic systems that are present under all conditions but typically buffered during steady-state physiology [15]. Three broad classes of constraint-shifted states illustrate this principle.

### 7.3. Signaling-Driven Flux Redistribution

In proliferative or growth-activated states, signaling pathways modulate nutrient uptake, redox balance, and ATP demand simultaneously. Such coordinated shifts reallocate flux across metabolic networks without requiring proportional enzyme abundance changes [62,63].

For example, activation of growth-promoting signaling cascades can increase glucose uptake, alter mitochondrial coupling efficiency, and reshape NAD^+^/NADH ratios. These changes expand substrate availability and shift elasticity profiles, enabling enhanced glycolytic throughput even when enzyme levels change modestly.

Importantly, elevated lactate or altered TCA intermediate levels in such contexts do not necessarily indicate simple overexpression of pathway enzymes. Instead, they reflect redistributed flux under modified thermodynamic and redox constraints.

Mutations in metabolic enzymes further demonstrate how small catalytic alterations can generate disproportionate metabolomic consequences. Altered enzyme specificity may lead to the accumulation of metabolites that subsequently influence epigenetic or signaling networks. Here, metabolite-driven feedback precedes broader proteomic remodeling [49,64]. These signaling-driven regimes illustrate how catalytic capacity and realized metabolic state can diverge when network context is redefined.

### 7.4. Redox-Repositioned States

Cells frequently encounter situations in which electron acceptor availability or mitochondrial efficiency changes. When NADH oxidation is limited, redox ratios shift rapidly, propagating through dehydrogenase reactions and altering pathway directionality [21,65,66,67].

Elevated NADH/NAD^+^ ratios can slow TCA turnover through product inhibition, redirect pyruvate toward lactate to regenerate NAD^+^, reconfigure shuttle systems, and induce compensatory anaplerotic inputs. In addition, pyruvate dehydrogenase (PDH) is regulated through PDK-mediated phosphorylation rather than simple product inhibition, further contributing to flux suppression under high NADH/NAD^+^ conditions [21]. These adjustments arise from redox constraints rather than large-scale abundance remodeling.

Steady-state metabolite pools under such conditions may increase despite reduced flux, or remain stable despite altered throughput, depending on downstream bottlenecks and compensatory routing. Pool–flux dissociation becomes especially apparent in these redox-dominated regimes.

Such states are observed during hypoxia, rapid immune activation, mitochondrial perturbation, and metabolic stress. The common feature is constraint repositioning, not necessarily enzyme deficiency.

### 7.5. Feedback-Amplified Transitional States

Across signaling-driven, redox-repositioned, and feedback-amplified states, a common pattern emerges: catalytic capacity changes slowly, constraint structure can shift rapidly, flux redistributes before abundance remodeling, and metabolite pools reflect balance under new constraints. These regimes do not represent pathological exceptions. They make visible a structural principle that applies broadly: enzyme abundance defines potential, whereas metabolic state is selected within a constraint-governed network space [13,15,35].

Proteome–metabolome discordance, therefore, intensifies when systems transition between constraint configurations. Rather than treating such a mismatch as an inconsistency, it should be interpreted as evidence of a state transition.

Some biological contexts involve rapid activation of metabolite-mediated feedback loops, in which accumulation of specific intermediates modifies transcription factors or regulatory enzymes [46,49,68].

## 8. Constraint-Based Modeling and Interpretative Framework

**Figure 3:**
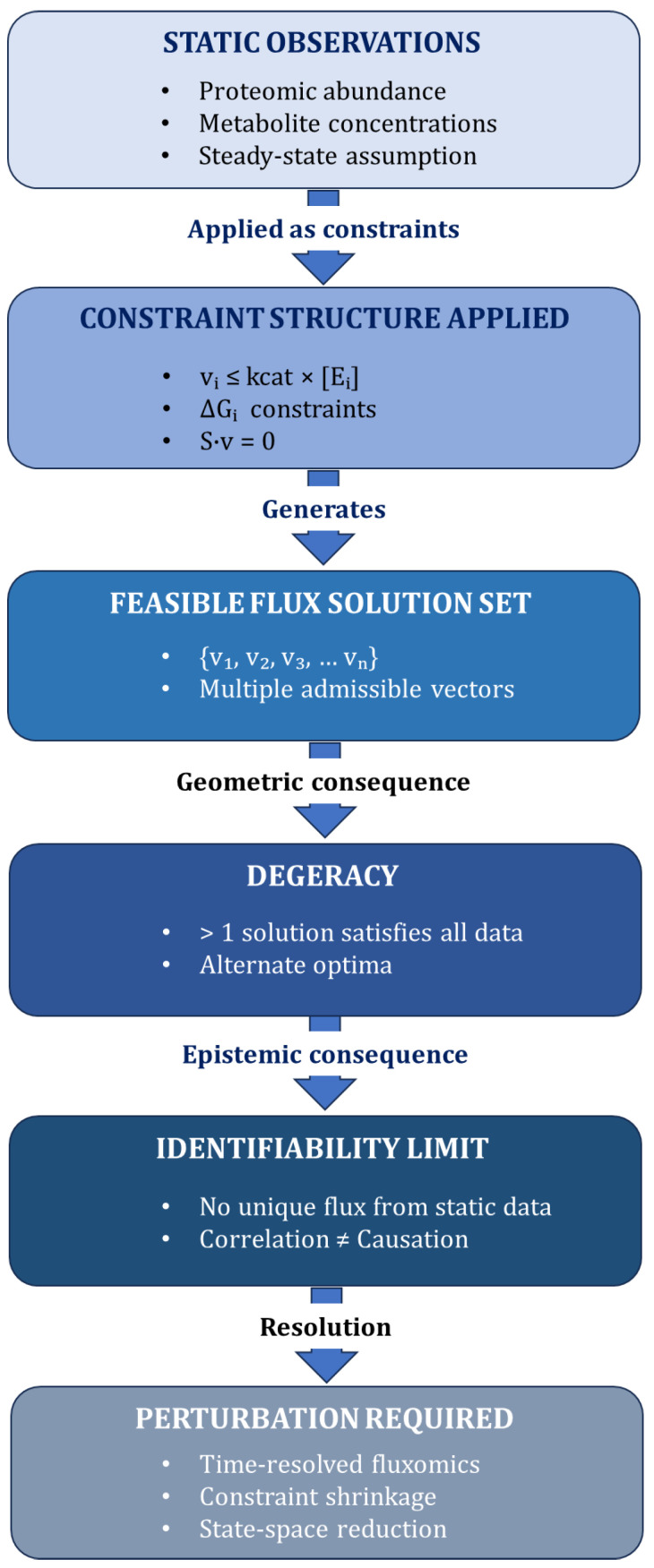
Identifiability problem arising from degenerate constraint structure. Conceptual framework illustrating why static multi-omic measurements are insufficient to uniquely infer metabolic flux states.

Metabolic networks are inherently underdetermined, allowing multiple flux distributions to satisfy identical proteomic and metabolomic states. As a result, static correlation between enzyme abundance and metabolite levels is insufficient for mechanistic inference. Constraint-based modeling approaches, such as flux balance analysis (FBA), provide a formal framework for describing metabolic networks [16]. In these models, metabolic fluxes are represented as a vector v constrained by the stoichiometric matrix S, such that S · v = 0 under steady-state assumptions. Additional constraints, including reaction bounds and thermodynamic directionality, further restrict the feasible solution space. To obtain a specific flux distribution, an objective function (e.g., biomass production or ATP maximization) is typically defined and optimized within this constrained space. Importantly, multiple feasible solutions may satisfy the same constraints, highlighting the inherent degeneracy of metabolic network states. This conceptual degeneracy is illustrated in Figure 3.

**Figure 4:**
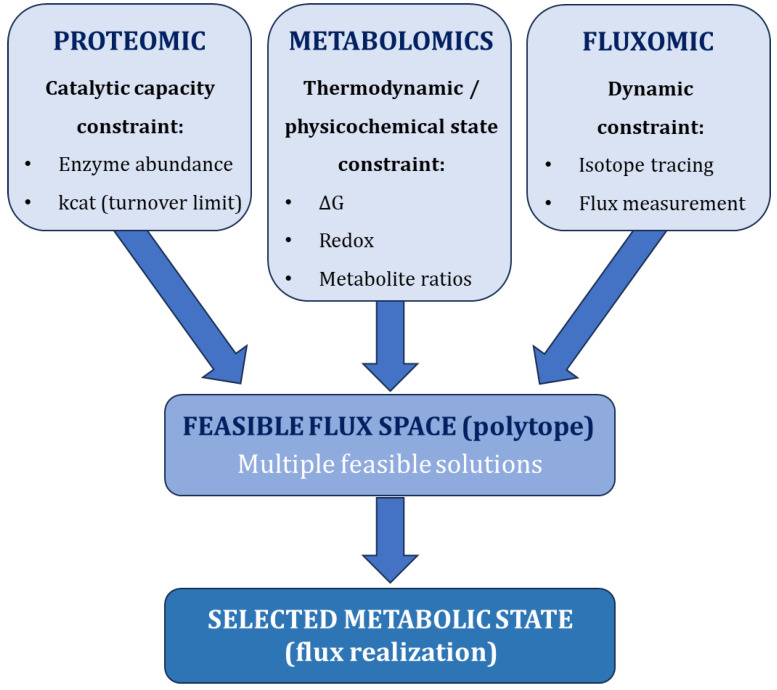
Integrated constraint-based framework for multi-omics interpretation. Proteomics constrains catalytic capacity, metabolomics informs reaction directionality, metabolite pool bounds, and physicochemical state (e.g., redox balance), and fluxomics constrains dynamic pathway activity. The intersection of these constraints defines a feasible flux space (polytope) containing multiple admissible solutions, within which biological systems select context-dependent metabolic states. This framework explains why static cross-omic correlation is insufficient for mechanistic inference.

Within this framework, proteomic data impose upper bounds on catalytic capacity, while metabolomic data constrain thermodynamic feasibility. However, these constraints alone do not uniquely determine flux distributions, as multiple feasible solutions remain within the constrained space (Figure 4; Table 2).

Fluxomics provides a critical bridging layer, although it encompasses multiple distinct methodological frameworks, including 13C-based metabolic flux analysis, dynamic (non-steady-state) flux analysis, and constraint-based approaches that incorporate flux measurements [69]. These approaches impose constraints through different mathematical formalisms and reduce solution degeneracy by providing additional information on pathway activity. By integrating these complementary layers, constraint-based modeling enables a more mechanistic interpretation of proteome–metabolome relationships beyond static correlation. While quantitative modeling approaches provide deeper mechanistic resolution, their application often requires system-specific assumptions and parameterization, highlighting the need for integrative yet interpretable frameworks [16].

Proteomic abundance and metabolite concentrations, when applied as capacity, thermodynamic, and stoichiometric constraints, define a feasible solution set containing multiple admissible flux vectors. This geometric degeneracy implies that more than one flux solution can satisfy identical static data, leading to an identifiability limit in which correlation does not imply causation. Resolution requires perturbation-based approaches, including time-resolved fluxomics, constraint shrinkage, and reduction in the feasible state space.

## 9. Conclusions and Perspectives

Proteome–metabolome decoupling can be understood as a consequence of constraint-driven organization in metabolic networks rather than a simple breakdown of molecular correspondence. This review has highlighted that enzyme abundance defines catalytic capacity, whereas metabolic state is selected within a constrained solution space shaped by thermodynamic, redox, and regulatory factors.

By dissecting the mechanistic layers linking abundance, activity, flux, and metabolite pools, we demonstrate that static cross-omic correlations are inherently limited in their ability to capture system-level behavior. Instead, a constraint-based perspective provides a more coherent framework for interpreting multi-omics data by distinguishing between capacity, state, and flux.

Accordingly, integrating proteomics, metabolomics, and flux-informed analyses is essential to reduce ambiguity and improve mechanistic interpretability. Future progress will depend on combining perturbation-based designs, temporal resolution, and constraint-aware modeling approaches to better resolve causal relationships within metabolic systems.

## Figures and Tables

**Table 1:** Mechanistic Layers Contributing to Proteome–Metabolome Decoupling.

Layer	Biological Mechanism	Why Correlation Weakens	Key Reference
**Abundance → Activity**	Post-translational modification; proteoform heterogeneity	Activity ≠ total protein abundance	[17]
**Abundance → Activity**	Enzyme complex assembly/metabolons	Functional incorporation ≠ expression level	[18]
**Activity → Flux**	Distributed control (MCA)	No single enzyme uniquely determines flux	[5]
**Activity → Flux**	Elasticity and substrate dependence	Capacity ≠ realized throughput	[7]
**Flux → Pool**	Steady-state balance and turnover	Pool size ≠ flux magnitude	[19]
**Flux → Pool**	Thermodynamic buffering	Near-equilibrium ratios insensitive to abundance	[20]
**Global Constraints**	Redox and energy charge regulation	State variables override local abundance effects	[21]
**Feedback**	Metabolite-driven transcriptional/epigenetic regulation	Bidirectional causality	[22]
**Spatial**	Organelle compartmentalization	Bulk averaging obscures local coupling	[23]
**Temporal**	Asynchronous adaptation	Static sampling conflates regulatory phases	[24]

**Table 2:** Constraint-Based Interpretation Framework for Multi-Omics Integration.

Omics Layer	What It Constrains	What It Does NOT Uniquely Determine	Key Reference
**Proteomics**	Upper bounds on catalytic capacity (subject to regulatory modulation)	Realized flux distribution	[5]
**Metabolomics**	Reaction directionality, metabolite pool bounds, and physicochemical state (including redox balance)	Unique flux magnitude	[20]
**Fluxomics**	Dynamic pathway throughput	Objective function or full network state	[69,70]
**Stoichiometric Modeling**	Feasible solution space (S · v = 0)	Single causal solution	[15]
**Perturbation Experiments**	Directionality of control	Steady-state uniqueness	[71,72]
**Statistical Correlation**	Cross-layer association	Mechanistic causality	[72]

## Data Availability

No new data were created or analyzed in this study. Data sharing is not applicable to this article.

## Abbreviations

ADP	Adenosine Diphosphate
AMP	Adenosine Monophosphate
ATP	Adenosine Triphosphate
ΔG	Gibbs Free Energy Change
FBA	Flux Balance Analysis
Gibbs free energy (ΔG°’)	Standard Transformed Gibbs Free Energy
kcat	Turnover Number
MCA	Metabolic Control Analysis
NAD^+^	Nicotinamide Adenine Dinucleotide (oxidized form)
NADH	Nicotinamide Adenine Dinucleotide (reduced form)
NADP^+^	Nicotinamide Adenine Dinucleotide Phosphate (oxidized form)
NADPH	Nicotinamide Adenine Dinucleotide Phosphate (reduced form)
PTM	Post-Translational Modification
Q	Reaction Quotient
ROS	Reactive Oxygen Species
RT	Product of the gas constant and temperature
SCM	Structural Causal Model
S · v = 0	Stoichiometric Mass Balance Equation
TCA cycle	Tricarboxylic Acid Cycle
